# Supplementary material for: Structural basis for IL-33 recognition and its antagonism by the helminth effector protein HpARI2
Source: Nat Commun. 2024 Jun 19;15:5226. doi: 10.1038/s41467-024-49550-0 (PMC11189471; doi:10.1038/s41467-024-49550-0)
Supplement: Supplementary file 1 — Supplementary Information [file 41467_2024_49550_MOESM1_ESM.pdf]

**Supplementary Information**

# Structural basis for IL-33 recognition and its antagonism by the helminth effector protein HpARI2

Abhishek Jamwal, Florent Colomb, Henry J. McSorley and Matthew K. Higgins

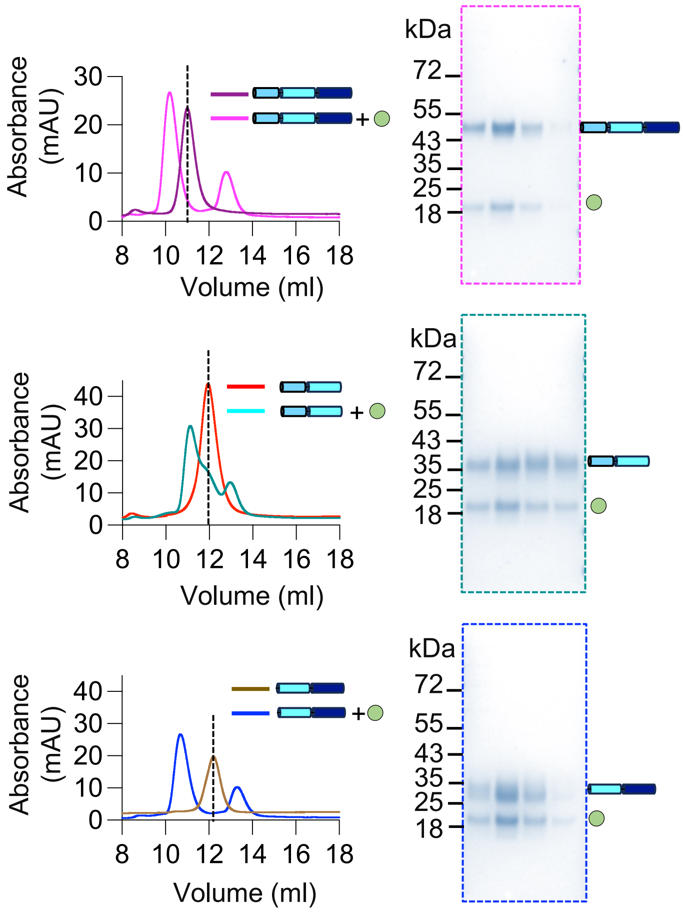


***Supplementary Figure 1: Assessment of formation of HpARI:IL-33 complexes***

The left panels show SEC traces of HpARI2 (top), HpARI2_CCP1/2 (middle) and HpARI2_CCP2/3 (lower), alone and in complex with mIL-33. The green circle represents IL-33 and the cylinders represent HpARI with CCP1 in mid-blue, CCP2 in lighter blue and CCP3 in dark blue. The respective SDS-PAGE are shown in the right panels. Molecular weights in kDa are marked on the gels.

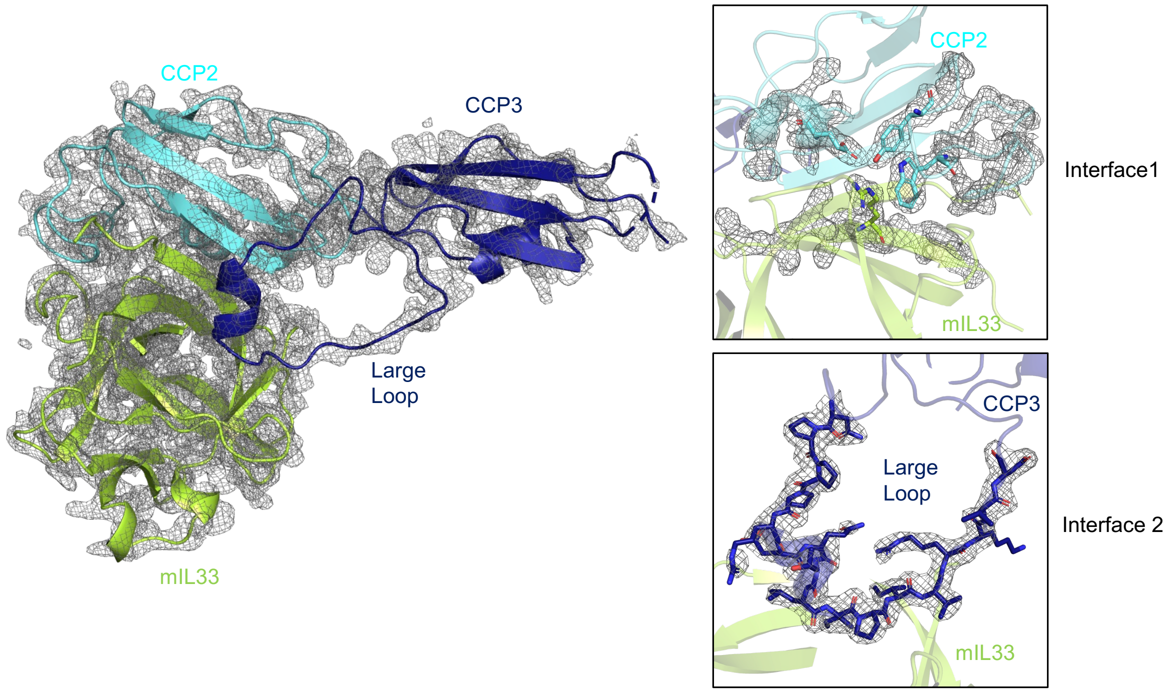


***Supplementary Figure 2: Electron density for the HpARI2:IL-33 complex***

The left panel shows the biological assembly of the HpARI2_CCP2/3: mIL-33 complex surrounded by electron density. The right panel shows the binding interfaces of the complex with key residues and structural feature surrounded by electron mesh. The electron density is from the $2F_o$-$F_c$ map, shown as a grey mesh and contoured at 1.5 sigma level and carved at 2 sigma.

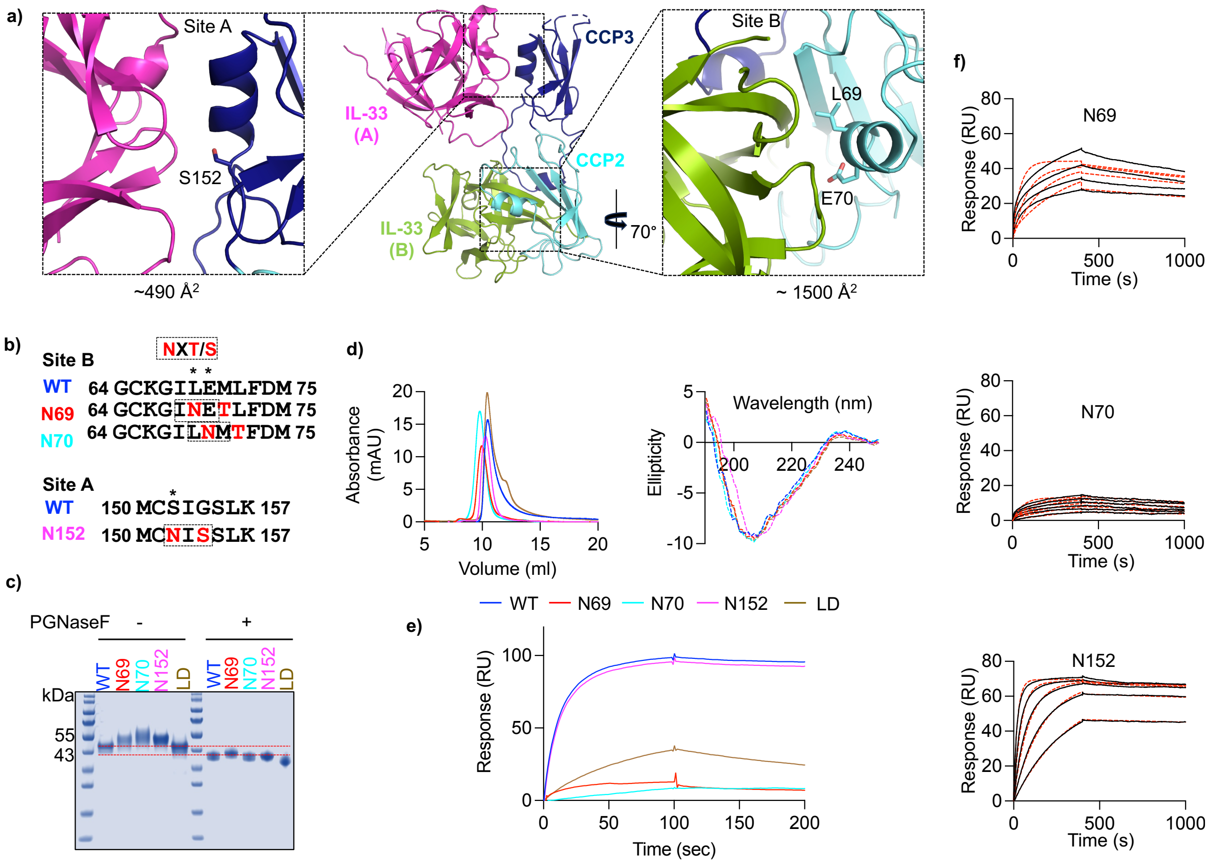


***Supplementary Figure 3: Determining which crystal contacts represent the physiological HpARI2_CCP2/3:mIL-33 complex***

**a)** Within the crystal, two IL-33 molecules contact each HpARI2, one through site A (left panel) and one through site B (right panel). Glycosylation sites were introduced to the residues indicated with sticks. **b)** Sequences of mutant forms of HpARI2 modified to add glycosylation sites, with glycans added to the residues marked with *. **c)** Assessment of HpARI2 and the glycosylation and loop deletion mutants on a Coomassie-stained gel, with and without treatment with PNGase F to remove glycans. **d)** Assessment of HpARI2 and the glycosylation and loop deletion mutants by size exclusion chromatography traces (left) and circular dichroism measurements (right). **e)** Surface plasmon resonance analysis in which 100 nM of HpARI2 and mutants were flowed over a mIL-33-coated surface. **f)** Surface plasmon resonance analysis (n=2) in which HpARI2 glycosylation mutants were injected over a mIL-33-coated surface, using two-fold dilution series from 4 μM for N69 and N70 and from 100 nM for N152. Solid black lines show response data and dashed red lines show fitting to a 1:1 binding model.

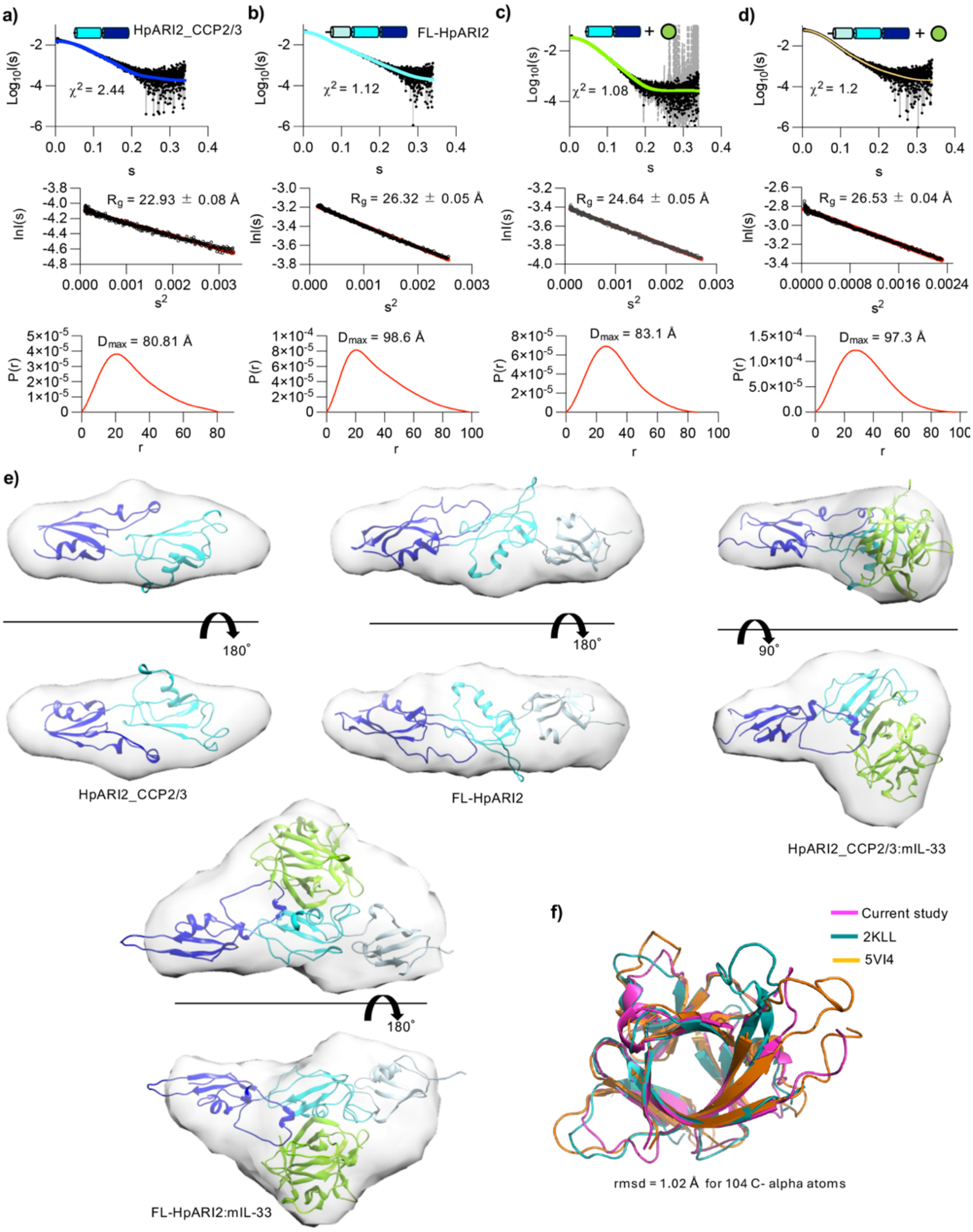


***Supplementary Figure 4: SAXS analysis of FL-HpARI2, HpARI2-CCP2/3 and their complexes with IL-33***

SAXS data for **a)** HpARI2_CCP2/3, **b)** FL-HpARI2, **c)** HpARI2_CCP2/3 bound to IL33 and **d)** FL-HpARI2 bound to IL-33. In each case, the upper panel shows a buffer subtracted scattering plot (black circles), with fitted theoretical curves from either crystal structures or Alphafold2 models (dark blue, light blue, green or yellow lines respectively). The middle panels show Guinier plots and the lower panels show pair distribution function plots. **e)** Envelopes calculated from SAXS data into which we have docked crystal structure of HpARI2_CCP2/3 or HpARI2_CCP2/3:mIL-33 complex, or Alphafold 2 models of FL-HpARI2 and FL-HpARI2:mIL-33 complex. **f)** Alignment of mIL-33 molecules from this study (magenta), ST2-bound mIL-33 (orange; PDB: 5VI4 [https://doi.org/10.2210/pdb5VI4/pdb] (IL33:ST2:IL-1RAcP)) and unbound hIL-33 (green; PDB: 2KLL [https://doi.org/10.2210/pdb2KLL/pdb] (IL33)).

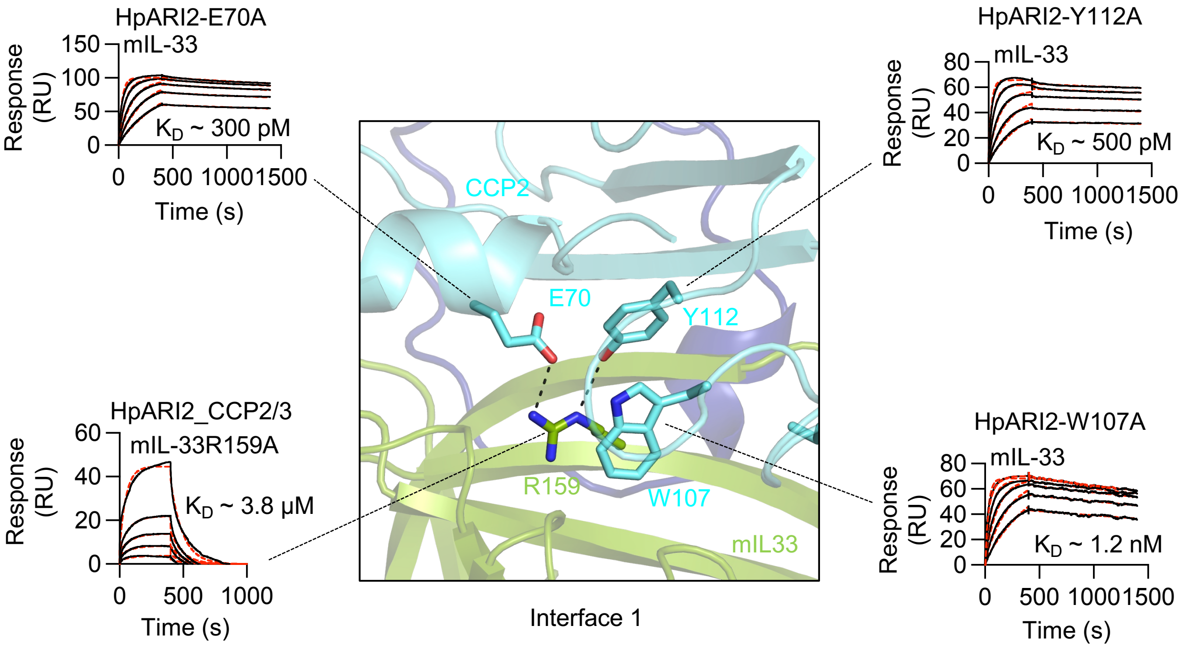


***Supplementary Figure 5: Analysis of the HpARI2_CCP2/3:mIL-33 interface by mutagenesis***

The central panel shows a view of interface 1 with residues targeted for mutational analysis shown as sticks. Surface plasmon resonance profiles are then shown for single site mutants of HpARI2 or mIL-33. Solid black lines show binding responses, whereas dashed red lines depict Langmuir fits. The SPR profiles for HpARI2 point mutants were generated by injecting a two-fold concentration series of mIL-33 starting at 1 µM, whereas a two-fold dilution series of mIL-33 R159A (starting at 4 µM) was applied to generate the kinetic profile for HpARI2_CCP2/3. Each SPR experiment was done twice (n=2).

```
a) hIL-33      SSITGISPITEYLASLSTYNDQSITFALEDESYEIYVEDLKKDEKKDKVLLSYYESQHPS 166
   mIL-33      -SIQGTSLLTQSPASLSTYNDQSVSFVLENGCYVINVDDSGKDQEQDQVLLRYYESPCPA 167
                ** * * :*:  **********::*.**: .* * *:*  **:::*:*** ****  *:

   hIL-33      NESGDGVDGKMLMVTLSPTK--DFWLHANNKEHSVELHKCEKPLPDQAFFVLHNMHSNCV 228
   mIL-33      SQSGDGVDGKKVMVNMSPIKDTDIWLHANDKDYSVELQRGDVSPPEQAFFVLHKKSSDFV 227
               .:******** :**.:** *  *:*****:*::****:: :   *:*******:  *: *

   hIL-33      SFECKTDPGVFIGVKDNHLALIKVDSSENLCTENILFKLSET 270
   mIL-33      SFECKNLPGTYIGVKDNQLALVEEKDE---SCNNIMFKLSKI 266
                *****. **.:******:***:: ...   . :**:****:
```

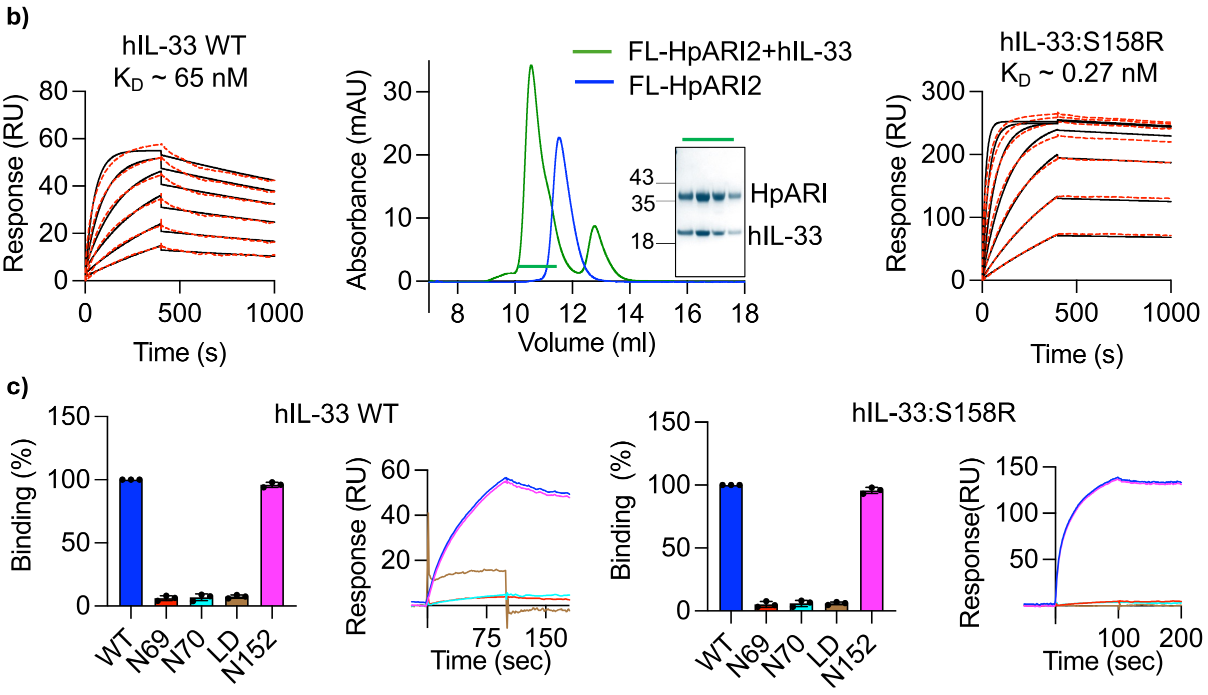


***Supplementary figure 6: Binding of HpARI2 to human IL-33***

**a)** Sequence alignment of mouse and human IL-33. Residues on mIL-33 which contact HpARI2_CCP2/3 are highlighted in green. **b)** SPR analysis of hIL-33 binding to biotinylated FL-HpARI2 (left panel) showing a two-fold dilution series from a maximum concentration of 1mM. Size exclusion mobility profile for FL-HpARI2, alone and bound to hIL-33 (central panel). SPR analysis of hIL-33:S158R binding to FL-HpARI2 showing a two-fold dilution series from a maximum concentration of 1mM (right panel). **c)** Binding of hIL-33 to HpARI2 variants determined by surface plasmon resonance analysis at a hIL-33 concentration of 1000 nM. Bars represent the average and standard deviation for three independent measurements (n=3).

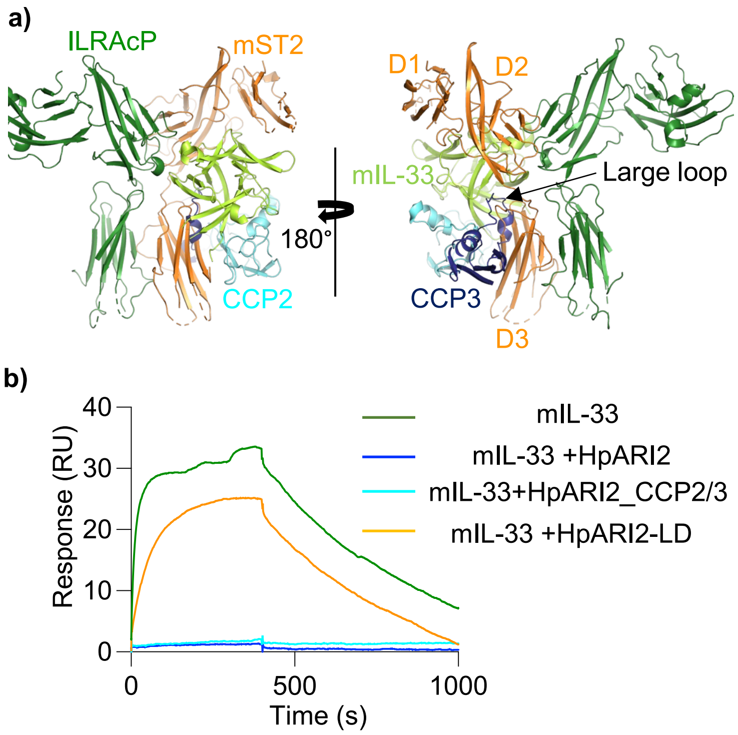


***Supplementary Figure 7: Inhibition of IL-33 signalling complexes by HpARI2***

**a)** The crystal structure of HpARI2_CCP2/3:mIL-33 docked on that of the mIL-33:mST2:ILRAcP complex (5VI4, [https://doi.org/10.2210/pdb5VI4/pdb] (IL33:ST2:IL-1RAcP))[9]. **b)** Representative surface plasmon resonance traces assessing binding of mIL-33 to immobilised mST2-Fc, alone or in the presence of HpARI2 variants, with HpARI2 in blue, HpARI2_CCP2/3 in cyan, HpARI2-LD in orange and mIL-33 alone in green. Experiments were performed twice.

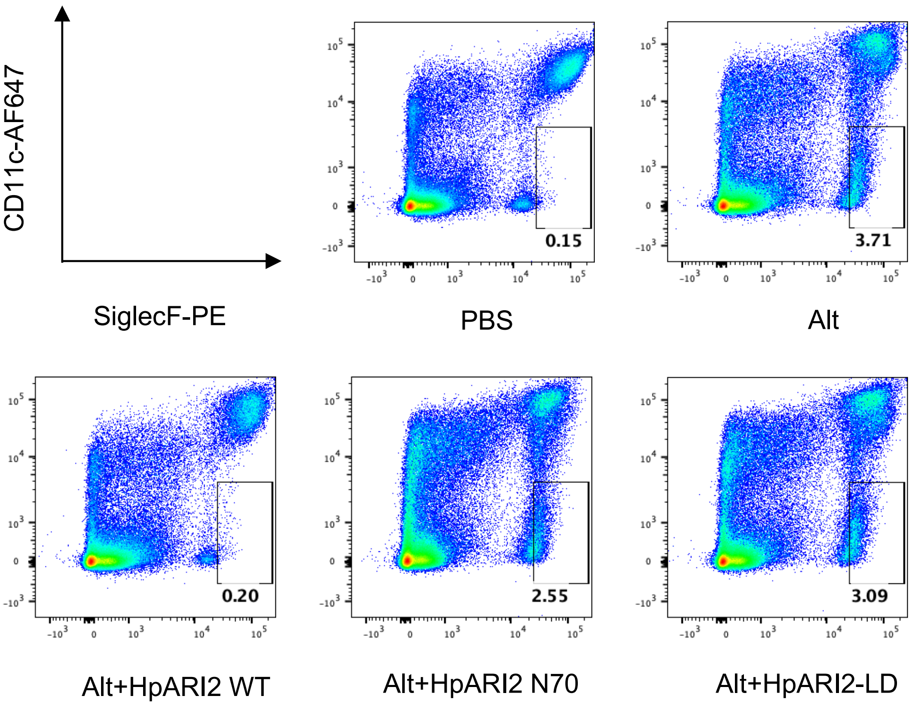


***Supplementary Figure 8: Representative flow cytometry staining for SiglecF and CD11c gated on live, CD45+ lung cells.***

***Supplementary Table 1: Surface Plasmon Resonance Binding constants derived for various HpARI2 mutants by fitting sensograms with Langmuir 1:1 model.***

| **Ligand** | **Analyte** | **$k_{ON}$ ($M^{-1}s^{-1}$)** | **$k_{OFF}$ ($s^{-1}$)** | **$K_D$ (M)** | **$R_{max}$ (RU)** | **$\chi^2$ ($RU^2$)** |
|---|---|---|---|---|---|---|
| HpARI2 | mIL-33 | $9.42 \times 10^5$ | $4.51 \times 10^{-5}$ | $4.78 \times 10^{-11}$ | 72.8 | 3.3 |
| HpARI2_CCP1/2 | mIL-33 | $4.08 \times 10^4$ | $2.01 \times 10^{-3}$ | $4.92 \times 10^{-8}$ | 189.1 | 7.9 |
| HpARI2_CCP2/3 | mIL-33 | $9.72 \times 10^4$ | $3.87 \times 10^{-4}$ | $3.98 \times 10^{-9}$ | 87.5 | 3.6 |
| HpARI2-LD | mIL-33 | $6.82 \times 10^4$ | $2.96 \times 10^{-3}$ | $4.53 \times 10^{-9}$ | 118 | 4.56 |
| HpARI2_CCP2/3 | mIL-33R159A | $2.037 \times 10^3$ | $7.86 \times 10^{-3}$ | $3.81 \times 10^6$ | 81 | 8.76 |
| HpARI2-E70A | mIL-33 | $3.19 \times 10^5$ | $9.89 \times 10^{-5}$ | $3.10 \times 10^{-10}$ | 78 | 4.8 |
| HpARI2-W107A | mIL-33 | $1.36 \times 10^5$ | $1.62 \times 10^{-4}$ | $1.18 \times 10^{-9}$ | 102 | 7.8 |
| HpARI2-Y112A | mIL-33 | $1.80 \times 10^5$ | $9.33 \times 10^{-5}$ | $5.28 \times 10^{-10}$ | 91 | 7.4 |
| HpARI2-N69 | mIL-33 | NA | NA | NA | NA | NA |
| HpARI2-N70 | mIL-33 | NA | NA | NA | NA | NA |
| HpARI2-N152 | mIL-33 | $1.29 \times 10^6$ | $6.12 \times 10^{-5}$ | $4.74 \times 10^{-11}$ | 72 | 2.79 |
| HpARI2 | hIL-33 | $6.42 \times 10^3$ | $3.65 \times 10^{-4}$ | $6.41 \times 10^{-8}$ | 74 | 8.1 |
| HpARI2 | hIL-33S158R | $1.20 \times 10^5$ | $3.33 \times 10^{-5}$ | $2.75 \times 10^{-10}$ | 255 | 18.9 |

*Supplementary Table 2: Crystallographic statistics for HpARI2_CCP2/3:mIL33 complex*

| | |
|---|---|
| **Data collection** | |
| Space group | C121 |
| a, b, c (Å) | 149.779, 35.754, 61.288 |
| α, β, γ (°) | 90, 92.07, 90 |
| Resolution (Å) | 48.26-2.1 (2.14-2.1) |
| Total observations | 131874 (6612) |
| Total unique | 19343 (1046) |
| $R_{pim}$ | 0.053 (0.663) |
| $CC_{1/2}$ | 0.998 (0.723) |
| Completeness (%) | 100 (100) |
| Multiplicity | 6.8 (7.0) |
| Wilson B factor ($Å^2$) | 47.5 |
| I/σ(I) | 10.4 (0.9) |
| **Refinement** | |
| Number of reflections | 19312 |
| Reflections in test set | 1542(151) |
| $R_{work}/R_{free}$ | 0.190/0.231 |
| Average B factor ($Å^2$) | 62.4 |
| Number of residues: | |
| Amino acid residues | 313 |
| Waters | 136 |
| RMSZ deviations: | |
| Bond lengths | 0.002 |
| Bond angles | 0.504 |
| Ramachandran plot | |
| Favoured (%) | 98.0 |
| Allowed (%) | 2 |
| Outliers (%) | 0 |

Values in the parenthesis are for the highest resolution shell.

***Supplementary Table 3: SEC-SAXS data table***

| | HpARI2_CCP2/3 | FL-HpARI2 | HpARI2_CCP2/3:mIL-33 | FL-HpARI2:mIL-33 |
|---|---|---|---|---|
| **Data collection parameters** | | | | |
| Instrument | Diamond Light Source (B21, Oxford) | | | |
| Wavelength (Å) | 0.9464 | | | |
| Energy (keV) | 13.1 | | | |
| Detector | Eiger X 4M (Dectris) | | | |
| Q range ($Å^{-1}$) | 0.0045 – 0.34 | | | |
| Detector distance (mm) | 3702.5 | | | |
| Protein concentration (mg/ml) | 7 mg/ml | 8 mg/ml | 8 mg/ml | 8 mg/ml |
| **Structural Parameters** | | | | |
| I(0) ($cm^{-1}$) | 0.018 | 0.043 | 0.033 | 0.063 |
| Rg (Guinier), (Å) | 22.93 | 26.32 | 24.64 | 26.53 |
| Rg (model) (Å) | 20.04 | 25.61 | 20.03 | 26.91 |
| Rg [P(r)], (Å) | 23.37 | 26.97 | 24.96 | 26.98 |
| $D_{max}$ (Å) | 80.81 | 98.6 | 83.13 | 97.3 |
| Porod volume($Å^{-3}$) | 16391 | 31476 | 32255 | 52080 |
| **Molecular Weight (kDa)** | | | | |
| $I_o$ | 16.52 | 21.78 | 31.64 | 39.09 |
| MoW | 19.26 | 24.97 | 36.97 | 44.28 |
| Vc | 17.841 | 22.12 | 33.12 | 39.86 |
| Size and shape | 21.72 | 23.94 | 37.86 | 42.36 |
| Primary sequence | 19.04 | 25.9 | 36.77 | 43.52 |
