# Supplementary material for: Structural basis for IL-33 recognition and its antagonism by the helminth effector protein HpARI2
Source: Nat Commun. 2024 Jun 19;15:5226. doi: 10.1038/s41467-024-49550-0 (PMC11189471; doi:10.1038/s41467-024-49550-0)
Supplement: Supplementary file 3 — Reporting Summary [file 41467_2024_49550_MOESM3_ESM.pdf]

nature portfolio

Corresponding author(s): Matthew K. Higgins and Henry J McSorley

Last updated by author(s): 30-4-24

Reporting Summary

Nature Portfolio wishes to improve the reproducibility of the work that we publish. This form provides structure for consistency and transparency in reporting. For further information on Nature Portfolio policies, see our Editorial Policies and the Editorial Policy Checklist.

Statistics

For all statistical analyses, confirm that the following items are present in the figure legend, table legend, main text, or Methods section.

n/a	Confirmed	
☐	☒	The exact sample size (*n*) for each experimental group/condition, given as a discrete number and unit of measurement
☐	☒	A statement on whether measurements were taken from distinct samples or whether the same sample was measured repeatedly
☐	☒	The statistical test(s) used AND whether they are one- or two-sided<br>*Only common tests should be described solely by name; describe more complex techniques in the Methods section.*
☒	☐	A description of all covariates tested
☒	☐	A description of any assumptions or corrections, such as tests of normality and adjustment for multiple comparisons
☐	☒	A full description of the statistical parameters including central tendency (e.g. means) or other basic estimates (e.g. regression coefficient) AND variation (e.g. standard deviation) or associated estimates of uncertainty (e.g. confidence intervals)
☒	☐	For null hypothesis testing, the test statistic (e.g. *F*, *t*, *r*) with confidence intervals, effect sizes, degrees of freedom and *P* value noted<br>*Give P values as exact values whenever suitable.*
☒	☐	For Bayesian analysis, information on the choice of priors and Markov chain Monte Carlo settings
☒	☐	For hierarchical and complex designs, identification of the appropriate level for tests and full reporting of outcomes
☒	☐	Estimates of effect sizes (e.g. Cohen's *d*, Pearson's *r*), indicating how they were calculated

Our web collection on statistics for biologists contains articles on many of the points above.

Software and code

Policy information about availability of computer code

Data collection	Data collection and processing software used are described in the methods section and are commercially available or openly accessible.
Data analysis	Data analysis was performed as described in the methods section using commercially available or openly accessible software. Software used for crystallographic data processing and structure determination is the standard in the field and is all freely available to academic users. GraphPad Prism version 10 was used to generate graphs, PyMOL for structure visualisation, and Affinity Photo and Designer and Adobe Photoshop and Illustrator to generate figures. BioEvaluation software is provided with the Biacore SPR machine and is the standard in the field.

For manuscripts utilizing custom algorithms or software that are central to the research but not yet described in published literature, software must be made available to editors and reviewers. We strongly encourage code deposition in a community repository (e.g. GitHub). See the Nature Portfolio guidelines for submitting code & software for further information.

Data

Policy information about availability of data

All manuscripts must include a data availability statement. This statement should provide the following information, where applicable:

- Accession codes, unique identifiers, or web links for publicly available datasets
- A description of any restrictions on data availability
- For clinical datasets or third party data, please ensure that the statement adheres to our policy

Coordinates and structure factors have been deposited in the Protein Data Bank with accession code 8Q5R. SAXS data is deposited in SASBDB with accession codes SAS5801-4.

Human research participants

Policy information about studies involving human research participants and Sex and Gender in Research.

Reporting on sex and gender	N/A
Population characteristics	N/A
Recruitment	N/A
Ethics oversight	N/A

Note that full information on the approval of the study protocol must also be provided in the manuscript.

Field-specific reporting

Please select the one below that is the best fit for your research. If you are not sure, read the appropriate sections before making your selection.

☒ Life sciences ☐ Behavioural & social sciences ☐ Ecological, evolutionary & environmental sciences

For a reference copy of the document with all sections, see nature.com/documents/nr-reporting-summary-flat.pdf

Life sciences study design

All studies must disclose on these points even when the disclosure is negative.

Sample size	Sample sizes are described in figure legends and methods. No statistical method was used to predetermine sample size. Quantitative experiments were typically repeated in technical triplicate.
Data exclusions	No data was excluded.
Replication	All SPR experiments were conducted at least 2 times. Alternaria in vivo mouse studies were performed two times and data pooled. All attempts at replication were successful
Randomization	No randomisation was conducted as no decisions about inclusion or exclusion of data were taken.
Blinding	No blinding was conducted as no decisions about data inclusion and exclusion were taken.

Reporting for specific materials, systems and methods

We require information from authors about some types of materials, experimental systems and methods used in many studies. Here, indicate whether each material, system or method listed is relevant to your study. If you are not sure if a list item applies to your research, read the appropriate section before selecting a response.

Materials & experimental systems

n/a	Involved in the study
☐	☒ Antibodies
☐	☒ Eukaryotic cell lines
☒	☐ Palaeontology and archaeology
☐	☒ Animals and other organisms
☒	☐ Clinical data
☒	☐ Dual use research of concern

Methods

n/a	Involved in the study
☒	☐ ChIP-seq
☐	☒ Flow cytometry
☒	☐ MRI-based neuroimaging

Antibodies

Antibodies used	Anti-mouse CD16/32 antibody (Biolegend, clone 93, #101302)<br>CD45-AlexaFluor700 (Biolegend, clone 30-F11, #103128)<br>Siglecf-PE (Miltenyi, clone ES22-10D8, #130-102-274)<br>CD11c-AlexaFluor647 (Biolegend, clone N418, #117312).
Validation	Commercially available antibodies.

Eukaryotic cell lines

Policy information about cell lines and Sex and Gender in Research

Cell line source(s)	Commercial Freestyle™ 293-F cells and Expi293F™ cells were used for recombinant protein expression.
Authentication	Cell lines were not authenticated except for recombinant expression of the target protein.
Mycoplasma contamination	All cell lines were not tested for mycoplasma contamination.
Commonly misidentified lines<br>(See ICLAC register)	No commonly misidentified cell lines were used in this work.

Animals and other research organisms

Policy information about studies involving animals; ARRIVE guidelines recommended for reporting animal research, and Sex and Gender in Research

Laboratory animals	In vitro experiments were performed using male C57BL/6J mice (Charles River). In vivo experiments were performed using female BALB/c purchased from Charles River (UK).
Wild animals	The study did not involve wild animals.
Reporting on sex	The study did not involve analysis based on sex
Field-collected samples	The study did not involve samples collected from the field.
Ethics oversight	Mice were accommodated and procedures performed under UK Home Office licenses with institutional oversight performed by qualified veterinarians. Home office project licence PP9520011.

Note that full information on the approval of the study protocol must also be provided in the manuscript.

Flow Cytometry

Plots

Confirm that:

☒ The axis labels state the marker and fluorochrome used (e.g. CD4-FITC).

☒ The axis scales are clearly visible. Include numbers along axes only for bottom left plot of group (a 'group' is an analysis of identical markers).

☒ All plots are contour plots with outliers or pseudocolor plots.

☒ A numerical value for number of cells or percentage (with statistics) is provided.

Methodology

Sample preparation	Murine lungs were digested in 2 U/ml of Liberase TL (Roche, Burgess Hill, UK) and 80 U/ml DNase (Life technologies, Paisley, UK) at 37°C with agitation for 35 min. Digested tissue was passed through a 70 mm strainer and red blood cells lysed using

ACK lysing Buffer (ThermoFisher). Live cells were counted using a haemocytometer and dead cells excluded using trypan blue. Single cell suspensions were washed in PBS and stained with Zombie Blue Live/Dead stain (Biolegend). Cells were then blocked with anti-mouse CD16/32 antibody (Biolegend) and surface stained.

Instrument	LSR Fortessa (BD Biosciences)
Software	FlowJo 10 (Treestar)
Cell population abundance	No sorting carried out.
Gating strategy	Cells were gated on live CD45+ cells, then on Siglecf-high, CD11c-negative eosinophils. Gating was based on unstained controls, apart from Siglecf staining which was gated related to PBS controls as a baseline, and SiglecF-high staining was gated based on a level greater than PBS controls.

☒ Tick this box to confirm that a figure exemplifying the gating strategy is provided in the Supplementary Information.
